# Prior knowledge guided eQTL mapping for identifying candidate genes

**DOI:** 10.1186/s12859-016-1387-9

**Published:** 2016-12-13

**Authors:** Yunli Wang, Rene Richard, Youlian Pan

**Affiliations:** 1 National Research Council Canada, 1200 Montreal Rd., Ottawa, K1A 0R6 Canada; 2 National Research Council Canada, 46 Dineen Dr., Fredericton, E3B 9W4 Canada

**Keywords:** eQTL mapping, Prior knowledge, Candidate genes, Lasso

## Abstract

**Background:**

Expression quantitative trait loci (eQTL) mapping is often used to identify genetic loci and candidate genes correlated with traits. Although usually a group of genes affect complex traits, genes in most eQTL mapping methods are considered as independent. Recently, some eQTL mapping methods have accounted for correlated genes, used biological prior knowledge and applied these in model species such as yeast or mouse. However, biological prior knowledge might be very limited for most species.

**Results:**

We proposed a data-driven prior knowledge guided eQTL mapping for identifying candidate genes. At first, quantitative trait loci (QTL) analysis was used to identify single nucleotide polymorphisms (SNP) markers that are associated with traits. Then co-expressed gene modules were generated and gene modules significantly associated with traits were selected. Prior knowledge from QTL mapping was used for eQTL mapping on the selected modules. We tested and compared prior knowledge guided eQTL mapping to the eQTL mapping with no prior knowledge in a simulation study and two barley stem rust resistance case studies.

The results in simulation study and real barley case studies show that models using prior knowledge outperform models without prior knowledge. In the first case study, three gene modules were selected and one of the gene modules was enriched with defense response Gene Ontology (GO) terms. Also, one probe in the gene module is mapped to Rpg1, previously identified as resistance gene to stem rust. In the second case study, four gene modules are identified, one gene module is significantly enriched with defense response to fungus and bacterium.

**Conclusions:**

Prior knowledge guided eQTL mapping is an effective method for identifying candidate genes. The case studies in stem rust show that this approach is robust, and outperforms methods with no prior knowledge in identifying candidate genes.

**Electronic supplementary material:**

The online version of this article (doi:10.1186/s12859-016-1387-9) contains supplementary material, which is available to authorized users.

## Background

A quantitative trait refers to a phenotype, such as disease resistance, that varies quantitatively and is attributable to multiple genes. The first step for discovering candidate genes is to identify chromosome regions associated with a particular quantitative trait through Quantitative trait loci (QTL) mapping. More recently, Expression quantitative trait loci (eQTL) mapping has been applied to identify regulatory regions for genes from transcriptome and genotype data. eQTLs are genomic loci that regulate expression in mRNAs or proteins. QTL mapping usually identifies a large region. Once QTLs or eQTLs have been identified, molecular techniques are employed to narrow down to candidate genes [1].

Traditional linkage mapping methods such as Haley-Knott regression(HK) and composite interval mapping (CIM) have been widely used for QTL mapping and recently on eQTL mapping [1]. Both HK and CIM assume that traits (QTL mapping) or genes (eQTL mapping) are not related. Association mapping methods based on the independence between genes and SNPs ignore the epistasis among genes and interaction between alleles. Least absolute shrinkage and selection operator (Lasso) generate a sparse regression model for eQTL mapping with one gene associated with a small number of SNPs. It showed that Lasso outperformed CIM and HK for eQTL mapping [2].

Recently, multi-task Lasso considering multiple correlated genes and multiple SNPs in a linear regression model has been used for eQTL mapping. Some methods have used prior knowledge to infer the associations between genes and SNPs [3]. The prior knowledge was either represented as gene pairs, SNP pairs, gene networks, or genetic interaction networks. Graph-guided fused lasso (GFLasso) used the fusion penalty to group related multi-response variables [4]. Adaptive Multi-Task Lasso assumed that genes are correlated and also used the prior knowledge of SNPs [5]. Chen et al. proposed a more efficient algorithm for GFLasso [6]. Fused Multitask Penalised Regression (FMPR) encourages the sparsity in weights for related tasks [7]. Two-graph guided multi-task Lasso allows the overlapped subnetworks of genes and SNPs, but they assume that correlation between SNPs or genes are known to infer the correlation between SNP and genes [8]. Graph-regularized dual Lasso represents the genetic interaction network and protein-protein interaction network as two graphs on a linear regression model [9]. Although these methods are appealing, they were only used on yeast and human eQTL mapping which contains rich biological knowledge.

Many eQTL mapping methods were used for identifying candidate genes [10–12]. Some used linkage mapping methods such as CIM [10, 11], and others used association mapping methods such as simple linear regression – Matrix eQTL [12], or GAPIT, single locus mapping for population structure [13]. However, most these studies using eQTL mapping for identifying candidate genes do not consider the correlated gene structure and genetic interactions between SNPs, and none of them used prior knowledge in eQTL mapping. Because such prior knowledge is usually not available or not reliable for most species, many studies still use the methods based on the independence assumption between gene and SNPs.

We proposed a new eQTL mapping method guided by prior knowledge for identifying candidate genes by using data-driven prior knowledge from QTLs/eQTLs in eQTL mapping. Although some advanced models [4, 5, 8, 9] are proposed and evaluated on model organism, the basic multi-task Lasso model has not been used for identifying candidate genes. We propose to use prior knowledge inferred from QTLs or eQTLs to set penalty factors for SNPs in multi-task Lasso. This method does not rely on any regulatory features of genes or SNPs. We compared eQTL mapping guided by prior knowledge with no prior knowledge. The results show that eQTL mapping guided by prior knowledge outperforms the model without knowledge. We applied our method on two case studies to identify candidate genes that are responsible for resistance to stem rust in barley.

## Methods

Our method has three steps. First, we perform QTL mapping to identify a relatively large chromosomal region associated with traits. Second, gene modules significantly associated with traits are selected. Then, the prior knowledge guided eQTL mapping method is performed on selected gene modules. In the second step, weighted correlation network analysis (WGCNA) is used for finding clusters (modules) of highly correlated genes [14]. Gene modules significantly associated with traits are selected.

For multiple correlated genes in selected gene modules, we used the multiple-response linear regression model LassoM [15] for eQTL mapping. Throughout this article, we use the letter “M” following the original model to represent multi-response model.

Consider *K* genes *Y*= [ *Y*_1_,…,*Y*_*S*_]^*T*^∈*R* ^*S*×*K*^ for *S* samples, a linear regression model for the functional mapping from *M* SNPs to *K* gene is given as

1 $$ Y = \beta_{0}+ X\beta  $$

Where *β*_0_ is a vector, *β*∈*R* ^*M*×*K*^ is a coefficient matrix, and *X*∈*R* ^*S*×*M*^. The objective function of LassoM:

2 $$ min_{(\beta_{0},\beta \in {R}^{M \times K})} \frac{1}{2N} \sum\limits_{i=1}^{N}\|y_{i}-\beta_{0}-x_{i}\beta\|_{F}^{2} + \lambda\sum\limits_{j=1}^{m}\|\beta_{j}\|_{2}  $$

LassoM is the linear regression model which gives the minimum mean cross-validated error.

We applied LassoMP by using prior knowledge on the basic model LassoM to identify candidate genes. We use letter “P” to represent prior knowledge. LassoMP uses prior knowledge in the multi-response linear regression model. From previous QTL or eQTL mapping, we learn some SNPs are strong regulators for a trait. Each SNP has a penalty factor in LassoM. Let *p*_*j*_∈ [ 0,1] be the penalty factor for *j*th SNP. For a particular SNP found from experimental results or QTLs identified from multiple experiments, their evidence is considered strong and thus *p*_*j*_=0 (no penalty). Otherwise, we can set *p*_*j*_=0.5 if the evidence is derived from computational result only. Also, the elastic-net penalty *α* is used to get a linear combination of ∥*β*_*j*_∥_1_ and $\|\beta _{j}\|_{2}^{2}$. The objective function becomes:

3 $$ \begin{aligned} &min_{\left(\beta_{0},\beta \in {R}^{m \times K}\right)}\frac{1}{2N}\sum\limits_{i=1}^{N}\|y_{i}-\beta_{0}-x_{i}\beta\|_{F}^{2}\\ &\quad+ \lambda \sum\limits_{j=1}^{m}p_{j}\left[(1-\alpha)\|\beta_{j}\|_{2}^{2}+\alpha\|\beta_{j}\|_{1}\right] \end{aligned}  $$

From this model, we can derive three models: LassoM, multi-response ridge (RidgeM) and multi-response elastic net(elasticM), and they differ in elastic-net penalty *α*: Lasso (*α*=1), ridge (*α*=0) and elastic net (*α*=0.5). They can be combined with prior knowledge *p*_*j*_ to generate their prior knowledge on multi-response models: LassoMP, RidgeMP and elasticMP.

## Results

### Simulation study

We performed the simulation study to compare LassoM and LassoMP with two other multi-task Lasso methods GFLasso [6] and FMPR [7]. GFLasso and FMPR are implemented in the R package FMPR. To demonstrate the effect of using prior knowledge, we also compared LassoM and LassoMP with RidgeM, RidgeMP, elasticM, and elasticMP. They are implemented in the R package glmnet [16]. In RidgeMP, LassoMP and elasticMP, the penalty factor is set as *p*_*j*_=0.

#### Simulation data

The performance of these eight models are compared in four setups. We set the number of samples *N*=50, vary the number of predictors *X* as 100 and 500, and the number of response variables *Y* as 10 and 20. We generated 30 datasets for each setups and compared the average performance of these models on the generated data. The simulation data is generated using the same method in [8]. The correlation between genetic markers and between genes are simulated. We compared the performance of these models using the root-mean-squared errors (RMSE), areas under the precision and recall curve (AUC), and degree of freedom (DF). RMSE and AUC were used to compare the performance of regression models in [8, 9]. We also used the DF since it indicates the number of predictors in the regression model. In eQTL mapping, usually a small number of genetic markers are associated with genes, so lower DF means less number of genetic markers in the model. The model with lower RMSE, higher AUC, and a lower DF are preferred. For each of the 30 datasets in four setups, cross-validation is performed on eight models and the optimal parameters are chosen, the models based on the optimal parameters are used to calculate RMSE, AUC and DF using the R package ROCR [17].

#### Simulation results

**Fig. 1:**
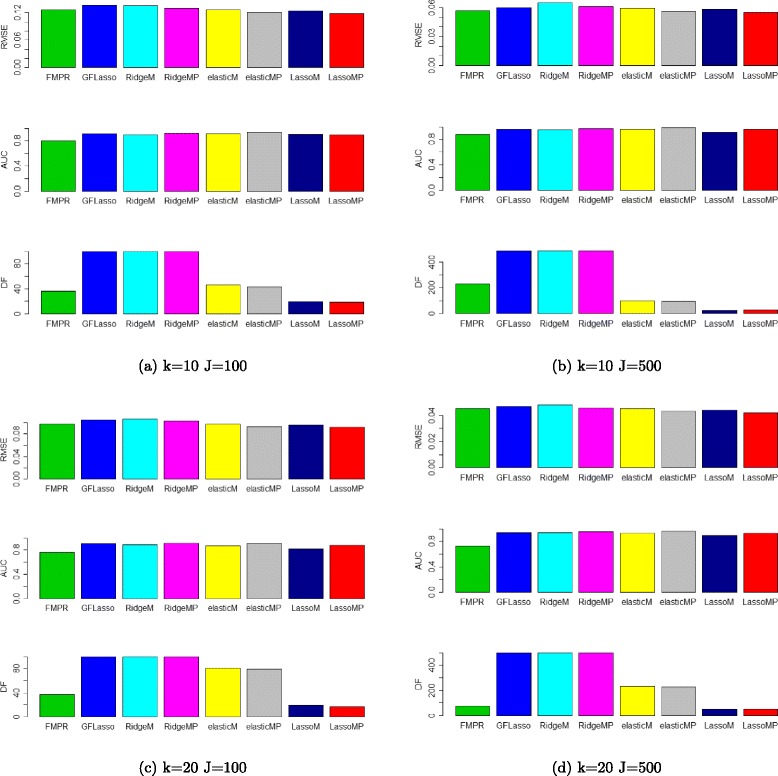
The performance of eight multi-response models in simulation study

The results of simulation study are shown in Fig. 1. Among eight models, LassoMP outperforms others in RMSE and DF, while elasticMP reaches the best performance in AUC. Specifically, LassoMP and LassoM outperform GFLasso and FMPR in RMSE and DF, and LassoMP performs better than FMPR but worse than GFLasso in AUC, but LassoM performs worse than GFLasso and FMPR in AUC.

Interestingly, the DFs of GFLasso, RidgeM and RidgeMP are always equal to the number of predictors, which means they used all predictors in the regression models. For eQTL mapping, if all genetic markers are included in a linear regression model, it might explain a large proportion of response and performs better in terms of AUC, but it violates the assumption that genes are regulated by a small number of genetic markers. Also, the number of predictors used in GFLasso, RidgeM, RidgeMP, elasticM and elasticMP are far more than the number of samples, which means these models are over-fitting. In all four setups, the number of predictors in LassoMP, LassoM and FMPR are always less than the number samples, and LassoMP achieved the lowest DF. LassoM and LassoMP are sparse models and thus need less numbers, yet most significant predictors as compared to other six models.

Comparing each pair of LassoMP vs. LassoM, RidgeMP vs. RidgeM, and elasticMP vs. elasticM, prior knowledge models reached better performance in AUC, RMSE and DF. It indicates that prior knowledge reduced cross-validation errors, predict accurately the associations between predictors and response variables using less number of predictors.

LassoMP achieved the lowest average RMSE and lowest DF among eight models in four setups. LassoMP reaches lower RMSE and comparable AUC with much less number of predictors than other models. From the simulation study, we conclude that LassoMP is a better model than others.

We also compared the computational time of eight models used in the simulation study. The average computational time of these models in five-fold cross validation of four experimental set ups are calculated (Table 1). LassoM, LassoMP, RidgeM, RidgeMP, elasticM, elasticMP are very efficient in all conditions. FMPR and GFLasso are more computationally expensive, and the runtime of GFLasso increases dramatically with the increase of number of response variables and predictors.

**Table 1:** The average computational time of eight models in four simulation set ups

Model	K=10 J=100	K=20 J=100	K=10 J=500	K=20 J=500
	(seconds)	(seconds)	(seconds)	(seconds)
FMPR	22.34	61.98	72.96	232.90
GFLasso	113.38	496.00	947.28	3776.94
RidgeM	0.44	0.82	0.79	1.67
RidgeMP	0.48	0.87	0.95	1.70
elasticM	0.47	0.89	0.62	1.67
elasticMP	0.48	0.95	0.61	1.70
LassoM	0.48	0.98	0.56	1.42
LassoMP	0.50	1.00	0.56	1.51

### Stem rust case study 1

In this study, we aim to perform prior knowledge guided eQTL mapping on a barley data set to identify candidate genes underlying resistance to the *Puccinia gramins f.sp. tritici* in barley. Rpg1 has been identified as the stem rust resistance gene in barley for many stem rust pathogen races [18].

#### Dataset

Our study used genotyptic and phenotypic data, and a genetic map of two barley populations “Steptoe” and “Morex” generated from [10]. The barley stem rust data sets were downloaded from Gene Network [19]. Barley phenotypic data were generated from a population of 150 F1-derived doubled haploid (DH) lines derived from the cross of Steptoe X Morex (St/Mx). Stem rust infection type was measured using numeric infection type scores [10]. The gene expression experiments were performed on a set of 139 lines of embryo-derived tissues in these 150 lines of barley [19]. The gene expression data set was derived from the Affymetrix Barley1 GeneChip, which contains 22841 probe sets. The barley genotype data set had 842 SNPs in each of 150 St/Mx DH lines. We imputed the missing data points in genotype data using PHASE [20]. After removing co-segregated SNPs, we collected 413 SNP markers from the genotype data. We removed 12 samples from genotype data and one sample from the gene expression data because of too many missing values in either genotype or gene expression data. The gene expression data set contains 22841 probe sets and 138 samples. More details of the original barley data can be found in [10].

#### QTL mapping

To identify candidate genes for resistance to stem rust, we used our three-step method: QTL mapping, gene module selection, and prior knowledge guided eQTL mapping.

QTL mapping is performed using the R/qtl R package [21]. All QTLs with LOD (logarithm (base 10) of odds) score at *p*-value <0.05 level in 1000 permutation tests are identified as QTLs from linkage mapping (Table 2). The only QTL revealed for stem rust infection type 0, 1, and 3 is located on chromosome 7H 0cM, which is co-located with Rpg1 SNP marker. One QTL on chromosome 2 at 49.3cM, co-located with ABC01899-1-1-301 SNP marker, is identified to be associated with stem rust infection type 2. The Rpg1 locus identified using linkage mapping in this data set coincides with the major stem rust resistance locus [18].

**Table 2:** QTLs for stem rust infection types

Trait	SNP	Chromosome	Centimorgan	LOD score
Stem Rust Type 0	Rpg1	7	0	46.14
Stem Rust Type 1	Rpg1	7	0	59.57
Stem Rust Type 2	ABC01899-1-1-301	2	49.3	3.29
Stem Rust Type 3	Rpg1	7	0	106.32

#### Gene module selection

Seventy-eight gene co-expression modules were generated using WGCNA. To summarize the gene expression profiles of the highly correlated genes inside a module, module eigengene (ME) is calculated based on the eigenvector of the first principle component of all genes in the module. Then, the correlation between each of stem rust infection type 0, 1, 2, 3 and each ME is calculated. If we consider stem rust infection type 0,1,2,3 as independent tests, which is a strict requirement, any module with *p*-value $<1.6 \times 10^{-4}\left (\frac {0.05}{78\times 4}\right)$ is considered as significantly correlated with traits. Three modules Plum1, Skyblue and Saddlebrown (Table 3) are significantly associated with stem rust infection type 0, 1, and 3.

**Table 3:** Gene modules significantly associated with stem rust infection types

Trait	Gene modules	Correlation coefficient (*p*-value)
Infection Type 0	Plum1	0.72 (2e-23)
	Saddlebrown	0.4 (9e-7)
	Skyblue	-0.7 (1e-21)
Infection Type 1	Plum1	0.79 (3e-30)
	Saddlebrown	0.45 (3e-8)
	Skyblue	-0,72 (1e-23)
Infection Type 2	None	
Infection Type 3	Plum1	-0.81 (1e-32)
	Saddlebrown	-0.45 (2e-8)
	Skyblue	0.76 (3e-27)

#### Prior knowledge guided eQTL mapping

At first, eQTL mapping between genes in selected gene modules and all SNP markers was performed using the basic model LassoM. Most genes in Plum1 and Skyblue gene modules both showed strong correlations with markers Rpg1 (7H@0cm) and Sft (7H@0.7cM), but genes in the Saddlebrown module had correlations with markers on 7H 28cM. The SNP markers for genes in the Plum1 and Skyblue modules overlap with the QTL identified in QTL mapping.

To evaluate LassoM and LassoMP, we compared them in terms of model selection measures: mean-squared error (MSE), and proportion of variance explained, and biological meaning measure: proportion of cis eQTL. The low mean-squared error or high proportion of variance explained indicates a better linear regression model. eQTLs that map to the approximate location of their gene-of-origin are referred to as cis eQTLs. In contrast, those that map far from the location of their gene-of-origin gene, often on different chromosomes, are referred to as trans eQTLs. The higher proportion of cis eQTL means the model identifies more eQTLs with SNPs located near associated genes. Usually the proportion of cis eQTL is used for measuring the biological meaning of eQTL mapping algorithms [2, 9].

**Fig. 2:**
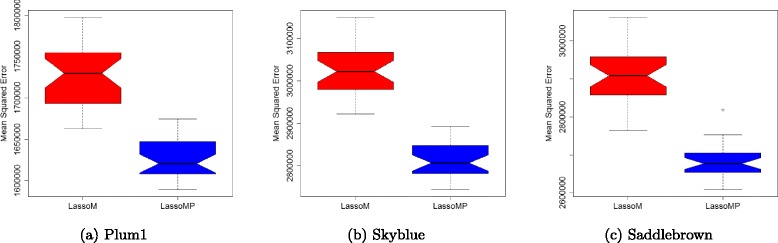
Comparison of LassoM and LassoMP on Plum1 (**a**), *Skyblue* (**b**), and *Saddlebrown* (**c**) gene modules

LassoM and LassoMP were compared on the same three selected gene modules: Plum1, Skyblue and Saddlebrown. The prior knowledge used in LassoMP on selected gene modules could be markers close to QTLs or eQTLs. Since Rpg1 is the QTL marker and also one of eQTL markers identified in Plum1 and Skyblue, we use Rpg1 as the prior knowledge for these two gene modules. For Saddlebrown, we used iEst5, 452-498, and 2124-984 SNP markers as the prior knowledge to guide the LassoMP process. The comparison between LassoM and LassoMP is performed in MSE by running 30 times five-fold cross validation (Fig. 2). The results show that LassoMP significantly outperforms LassoM on these three selected gene modules.

Next, we compare LassoM with LassoMP in terms of Mean-Squared Error and number of predictors (degree of freedom) in the linear regression models (Table 4). LassoM has lower mean-sqaured error. In all three gene modules, LassoMP only includes less then ten genetic markers in the model. It indicates that LassoMP achieves lower MSE with a much lower number of predictors in the model compared to LassoM. LassoMP has lower proportion of variance explained than that of LassoM because it uses less number of predictors. LassoMP are a better model than LassoM in terms of model selection.

**Table 4:** The comparison of four methods on three gene modules

Gene modules	Method	Mean	# of	Proportion	# of	# of eQTLs	Proportion
		Squared	predictors	of variance	eQTLs	with known	of cis eQTLs
		Error		explained(%)		gene locations	(%)
Plum1	LassoM	1750411	23	56.01	621	69	17.39
	LassoMP	1639614	9	52.13	243	27	29.63
Skyblue	LassoM	2918151	47	52.65	1269	47	9.57
	LassoMP	2801753	2	36.68	54	4	50.00
Saddlebrown	LassoM	2919870	14	46.73	378	28	21.43
	LassoMP	2675651	3	47.23	81	21	100.00

LassoMP are compared to LassoM in terms of the biological meaning (Table 4). The proportion of cis eQTLs identified from these four methods is used to measure the biological meaning. Less than 40% of probe sets in the Barley1 Affy platform are mapped to physical positions. We use 15 cM as the threshold for cis eQTL. The proportion of cis eQTLs from eQTL mapping of Plum1, Skyblue, Saddlebrown modules are shown in Table 4. LassoMP has a higher proportion of cis eQTLs than LassoM.

LassoMP is a better model than LassoM in terms of model selection and biological meaning. The cis eQTLs identified from LassoMP on Plum1, Skyblue and Saddlebrown gene modules are presented in Table 5. Probes with cis eQTLs and high correlation coefficients are potential candidate genes for resistance to stem rust, such as Contig3140_at, Contig6347_at, Contig2657_at, HVSMEn0014H06r2_s_at in Plum1, and Contig12634_at, Contig6348_at, Contig18035_at, Contig3141_at in Skyblue, and Contig40_x_at, Contig3269_at, Contig5614_s_at, Contig2957_at, and Contig4028_x_at in Saddlebrown.

**Table 5:** cis eQTLs identified in in Plum1, Skyblue and Saddlebrown modules

Modules	Probe sets	Chr	cM	SNP	Chr	cM
Plum1	AF509747.1_at	7	0.9	Rpg1	7	0.0
	Contig11996_s_at	7	12.7	Rpg1	7	0.0
	Contig9996_at	7	0.4	Rpg1	7	0.0
Skyblue	Contig14185_at	7	0.6	Rpg1	7	0.0
	Contig26418_at	7	0.2	Rpg1	7	0.0
Saddlowbrown	Contig10289_at	7	24.5	2124-984	7	29.2
	Contig11481_at	7	22.9	2124-984	7	29.2
	Contig11570_at	7	25.9	2124-984	7	29.2
	Contig13623_at	7	27.6	2124-984	7	29.2
	Contig18611_at	7	29.8	2124-984	7	29.2
	Contig5613_at	7	22.7	2124-984	7	29.2
	Contig6931_at	7	24.2	2124-984	7	29.2
	Contig10289_at	7	24.5	452-498	7	28.5
	Contig11481_at	7	22.9	452-498	7	28.5
	Contig11570_at	7	25.9	452-498	7	28.5
	Contig13623_at	7	27.6	452-498	7	28.5
	Contig18611_at	7	29.8	452-498	7	28.5
	Contig5613_at	7	22.7	452-498	7	28.5
	Contig6931_at	7	24.2	452-498	7	28.5
	Contig10289_at	7	24.5	iEst5	7	16.8
	Contig11481_at	7	22.9	iEst5	7	16.8
	Contig11570_at	7	25.9	iEst5	7	16.8
	Contig13623_at	7	27.6	iEst5	7	16.8
	Contig18611_at	7	29.8	iEst5	7	16.8
	Contig5613_at	7	22.7	iEst5	7	16.8
	Contig6931_at	7	24.2	iEst5	7	16.8

#### Validation

We used the alignment between the Affymetrix GeneChip Barley Genome Array and high confidence barley ensemble gene IDs provided by Ensemble Plants [22]. Based on these ensemble gene IDs, the GO annotations are retrieved from Biomart [23]. In each gene module, the GO enrichment analysis is performed using hyperGTest function in GOstats [24]. Enriched GO terms in Plum1 (Table 6) and in Skyblue modules (Additional file 1) reveals defense related functions. Plum1 includes three defense response probe sets: AF509747.1_at, HVSMEl0003E22r2_at and HS16G07u_at. AF509747.1_at represents the Rpg1 gene [10], which is a gene specific to stem rust in barley. HVSMEl0003E22r2_at and HS16G07u_at both link to the disease resistance related function–ADP binding. The Plum1 gene module is enriched with defense response, ADP binding, and cell wall related fructosyltransferase activity.

**Table 6:** Enriched GO terms of matched ensemble genes from probe sets in Plum1 module

GO	GOID	*P*value	Term
MF	GO:0047207	0.001	1,2-beta-fructan 1F-fructosyltransferase activity
MF	GO:0050738	0.002	fructosyltransferase activity
MF	GO:0043531	0.002	ADP binding
MF	GO:0090599	0.007	alpha-glucosidase activity
MF	GO:0004564	0.007	beta-fructofuranosidase activity
MF	GO:0004575	0.007	sucrose alpha-glucosidase activity
MF	GO:0015926	0.009	glucosidase activity
MF	GO:0005516	0.029	calmodulin binding
MF	GO:0070001	0.039	aspartic-type peptidase activity
MF	GO:0004190	0.039	aspartic-type endopeptidase activity
BP	GO:0070417	0.002	cellular response to cold
BP	GO:0034605	0.004	cellular response to heat
BP	GO:0046685	0.004	response to arsenic-containing substance
BP	GO:0006986	0.013	response to unfolded protein
BP	GO:0035967	0.013	cellular response to topologically incorrect protein
BP	GO:0034620	0.013	cellular response to unfolded protein
BP	GO:0006952	0.020	defense response
BP	GO:0006950	0.022	response to stress
CC	GO:0009506	0.024	plasmodesma
CC	GO:0030054	0.024	cell junction
CC	GO:0055044	0.024	symplast
CC	GO:0005911	0.024	cell-cell junction
CC	GO:0005783	0.038	endoplasmic reticulum

The Skyblue and Plum1 modules are associated to the same genetic marker Rpg1, but they show distinct functional annotations. Two probe sets Contig8651_at and Contig8651_s_at link to ADP-sugar diphosphatase activity, ADP-ribose pyrophosphohydrolase activity, ADP-glucose pyrophosphohydrolase activity, and one probe set Contig2598_s_at shows response to sucrose, glucose, and fructose. Skyblue is also enriched with transmembrane transport.

### Stem rust case study 2

#### Dataset

The second case study on stem rust resistance investigated the resistance to stem rust pathogen Ug99 in progeny of Q21861 and SM89010 [11]. Previous studies identified a major QTL Rpg-TTKSK on 5H in seedlings samples and other loci in adult plants [25]. The experiment was designed to examine the qualitative and quantitative resistance in seedlings and adult plants in response to Pgt race TTKSK. Crossing Q21861 and SM89010 generated 75 double haploid lines and each line has one sample treated with Pgt race TTKSK-inoculation (TTKS) and another sample treated with mock-inoculation (MOCK). The trait data includes infection types and infection severity in seedlings and adult plants. The gene expression data contains 22841 probesets from four biological replicates for each parental line, and 75 TTKS samples and 75 MOCK samples in QSM lines. Genotypic data uses the QSM genetic map from transcript-derived markers (TDMs), which include 378 markers. The gene expression data was downloaded from GEO [26], and phenotype and genotype data were downloaded from [11].

#### QTL mapping

In the second case study, we use the same three-step prior knowledge guided eQTL mapping method as above to identify candidate genes. Phenotype data used for QTL mapping was split into four categories: infection frequency (IF), principal component (PC), severity (SEV), and lesion size (LES), and infection coefficient (IC). IF and PC traits are phenotype data for seedling, and other two for adult plants. PC1, 2, 3, and 4 are derived from IF0, 1, 2, 3 for infection types 0, 1, 2, 3, and infection coefficient are derived from severity and lesion size. The genotypic data has 378 markers on 75 DH lines. R/qtl [21] was used for linkage mapping between phenotype and genotype (Table 7). From seedling samples, 5H@147cm is a major QTL, and some other QTLs are identified from 3H. From adult plants, 5H@141cm is the main QTL for SEV, LES and IC, a few QTLs on 7H and 2H are also identified.

**Table 7:** QTLs for stem rust infection in QSM population

Trait	Chromosome	Centimorgan	LOD score
IF for Infection Type 0	3H	0	4.06
IF for Infection Type 0	5H	147	19.36
IF for Infection Type 1	5H	147	16.5
IF for Infection Type 2	5H	145	3.28
IF for Infection Type 3	3H	6.8	4.49
IF for Infection Type 3	5H	146.8	21.97
PC1	3H	6.8	4.79
	5H	146.8	31.39
PC2	NULL		
PC3	NULL		
PC4	NULL		
SEV 7-Oct-08	5H	141.4	5.73
	7H	76.8	3.49
SEV 17-Oct-08	2H	41.5	2.97
	5H	141.4	6.31
SEV 10-Nov-08	5H	141	4.18
LSE 7-Oct-08	5H	141	7.3
LSE 17-Oct-08	3H	2.72	3.30
	5H	145.42	5.72
LSE 10-Nov-08	5H	72.2	4.71
IC 7-Oct-08	5H	141	6.54
IC 17-Oct-08	5H	141	6.88
IC 10-Nov-08	5H	141	4.2

#### Gene module selection

Since this dataset used TTKS and MOCK samples in pairwise experimental design, we first identified differentially expressed genes (DEG) from all samples. At first, we identified 362 DEGs from Q21861 and 4 DEGs from SM89010, merged them into 366 DEGs in parental lines with *p*-value <0.05. Clearly, Q21861 contributes much more DEGs than SM89010. Also, we identified 8460 DEGs from progeny among 75 paired samples with *p*-value <0.05. In total, 8487 DEGs were identified from parental lines and progeny.

WGCNA was used to generate gene modules from the 8487 DEGs which include 154 samples. These 154 samples include four samples from parental lines and 150 samples from 75 progeny lines in TTKS and MOCK conditions. Through the hierarchical clustering, 78 modules were generated from the DEGs. The gene modules that are significantly associated with a trait were selected. At first, gene expression values were transformed (TTKS gene expression values minus MOCK gene expression values) in 75 TTKS samples, and gene modules significantly associated with each trait were identified respectively.

The correlation coefficient between a gene module and a phenotype is considered significant with *p*-value <0.05. Four gene modules: Saddlebrown, Darkgrey, Darkmagenta, and Blue are significantly associated with infections in seeding and five gene modules Royalblue, Lightyellow, Yellow, Sienna3, and Darkgreen significantly associated with infections in adult plants (Table 8).

**Table 8:** Gene modules significantly associated with infection types in seedlings and adult plants

Trait		Gene modules	Correlation coefficient (*p*-value)
IF	IF0, IF3	Darkgrey	-0.23(0.0)
	IF2	Saddlebrown	-0.22(0.06)
PC	PC1	Darkgrey	-0.24(0.04)
	PC2	Saddlebrown	0.31(0.007)
	PC3	Darkmagenta	0.22(0.006)
	PC4	Darkmagenta	0.23(0.05)
		Blue	0.24(0.007)
SEV	SEV20081007	Royalblue	0.25(0.03)
		Lightyellow	0.27(0.02)
	SEV20081110	Royalblue	0.25(0.03)
		Lightyellow	0.27(0.02)
	SEV20081110	Royalblue	0.25(0.03)
LSE	LSE20081007	None	
	LSE20081017	Yellow	0.27(0.02)
	LSE20081110	Sienna3	0.24(0.04)
		Darkgreen	-0.31(0.006)
IC	IC20081007	Royalblue	0.25(0.03)
		Lightyellow	0.29(0.01)
	IC20081017	Royalblue	0.22(0.05)
		Lightyellow	0.28(0.02)
	IC20081110	None	

The Darkgrey and Saddlebrown modules are significantly associated with IF and PC. Darkgrey corresponds to PC1 and Saddlebrown associates with PC2. This indicates these two gene modules capture the main infection factors. Darkgrey is negatively associated with IF0 and IF3, and Saddlebrown is negatively associated with IF2. Since IF2 and IF3 represent high infection severity, the Darkmagenta and Blue modules correspond to PC3 and PC4, and it indicates that they capture minor factors for infection. Four gene modules are positively associated with SEV, LSE and IC, but only the Darkgreen is negatively associated with LSE.

#### Prior knowledge guided eQTL mapping

**Fig. 3:**
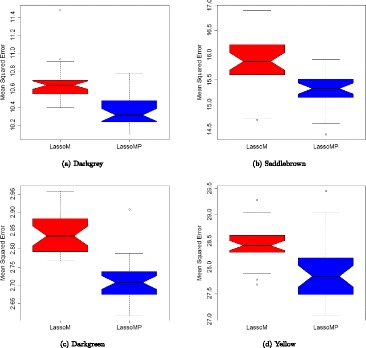
The comparison of LassoM and LasooMP on *Darkgrey* (**a**), *Saddlebrown* (**b**), *Darkgreen* (**c**), and *Yellow* (**d**) gene modules

We identified eQTLs in two gene modules, Saddlebrown and Darkgreen, using LassoM. Saddlowbrown is negatively associated with IF2 and Darkgreen with LSE. eQTL mapping is performed on Darkgrey, Saddlowbrown, Darkgreen and Yellow using LassoM and LassoMP. Subsequently, we compared LassoM and LassoMP on four gene modules: Darkgrey, Saddlebrown, Darkgreen, and Yellow using 5-fold cross validation (Fig. 3). Prior knowledge for each gene module using LassoMP is selected from candidate markers identified using LassoM, and the marker with lowest MSE are selected as the prior knowledge. In the Darkgrey module, 5H@144cm reached the lowest MSE, and overlaps with the QTL (5H Rpg-TTKSK QTL region) identified from QTL mapping (Table 7). The Darkgrey module corresponds to PC1 of infection types, which captures the main factor of infection in seedlings. In the Yellow module, LassoMP with genetic marker at 2H@153.5cm has a lower MSE. In other two gene modules Saddlowbrown and Darkgreen, LassoMP reaches lower MSE comparing to LassoM.

We used LassoMP as the mapping method on these four gene modules. Top 10 eQTLs identified from Darkgrey, Saddlowbrown, Darkgreen and Yellow gene modules are listed in Table 9.

**Table 9:** Top 10 eQTLs identified using LassoMPs on Darkgrey, Saddlowbrown, Darkgreen and Yellow gene modules

	Probes	SNP	Location	Correlation
				coefficient
Darkgrey	Contig3156_s_at	HZ58F11r_at	5H@145.4cm	0.505
	Contig3155_s_at	HZ58F11r_at	5H@145.4cm	0.350
	Contig1385_at	HZ58F11r_at	5H@145.4cm	0.264
	Contig3157_at	HZ58F11r_at	5H@145.4cm	0.262
	Contig19929_at	HZ58F11r_at	5H@145.4cm	0.259
	Contig3151_at	HZ58F11r_at	5H@145.4cm	0.256
	Contig11361_at	HZ58F11r_at	5H@145.4cm	0.196
	Contig8307_s_at	HZ58F11r_at	5H@145.4cm	0.147
	Contig6701_s_at	HZ58F11r_at	5H@145.4cm	0.143
	Contig5469_at	HZ58F11r_at	5H@145.4cm	0.108
Saddlebrown	Contig2210_at	Contig13249_at	7H@47.1cm	-0.642
	Contig2212_s_at	Contig13249_at	7H@47.1cm	-0.612
	Contig2209_at	Contig13249_at	7H@47.1cm	-0.411
	Contig2214_s_at	Contig13249_at	7H@47.1cm	-0.376
	HVSMEm0003C15r2_s_at	Contig13249_at	7H@47.1cm	-0.309
	Contig1637_s_at	Contig13249_at	7H@47.1cm	-0.288
	Contig1637_at	Contig13249_at	7H@47.1cm	-0.284
	Contig2787_s_at	Contig13249_at	7H@47.1cm	-0.284
	Contig13350_at	Contig13249_at	7H@47.1cm	-0.257
	EBem10_SQ002_I10_s_at	Contig13249_at	7H@47.1cm	-0.170
Darkgreen	Contig2170_at	Contig4572_at	7H@83.5cm	-0.807
	Contig8722_at	Contig4572_at	7H@83.5cm	-0.199
	Contig11240_at	Contig4572_at	7H@83.5cm	-0.149
	Contig21643_at	Contig4572_at	7H@83.5cm	-0.149
	Contig3886_at	Contig4572_at	7H@83.5cm	-0.144
	Contig23697_at	Contig4572_at	7H@83.5cm	-0.128
	Contig13049_at	Contig4572_at	7H@83.5cm	-0.106
	Contig13799_at	Contig4572_at	7H@83.5cm	-0.098
	Contig20_at	Contig4572_at	7H@83.5cm	-0.089
	Contig1315_s_at	Contig4572_at	7H@83.5cm	0.036
Yellow	Contig8002_at	Contig1791_x_at	2H@153.5cm	0.288
	Contig20602_at	Contig1791_x_at	2H@153.5cm	0.285
	Contig11328_at	Contig1791_x_at	2H@153.5cm	0.266
	Contig14754_at	Contig1791_x_at	2H@153.5cm	0.227
	Contig23817_at	Contig1791_x_at	2H@153.5cm	0.205
	Contig8052_at	Contig1791_x_at	2H@153.5cm	0.200
	Contig23584_at	Contig1791_x_at	2H@153.5cm	0.197
	HV_CEa0006L03r2_at	Contig1791_x_at	2H@153.5cm	0.190
	Contig10957_at	Contig1791_x_at	2H@153.5cm	0.188
	HA14H02r_at	Contig1791_x_at	2H@153.5cm	0.185

#### Validation

The enriched GO terms in Saddlebrown (Table 10), Darkgrey, Darkgreen gene modules (Additional file 2) are presented. Five probe sets (Contig2209_at, Contig2210_at, Contig2214_s_at, Contig2211_at, Contig2212_s_at) in the Saddlebrown gene module are mapped to the same UniProt gene Pathogenesis-related protein PRB1-2. Mapped ensemble genes are enriched with defense response to fungus and immune response.

**Table 10:** Enriched GO terms of matched ensemble genes from probe sets in Saddleborwn module

GO	GOID	*p*–value	Term
MF	GO:0008422	0.001	beta-glucosidase activity
MF	GO:0042973	0.001	glucan endo-1, 3-beta-D-glucosidase activity
MF	GO:0004553	0.001	hydrolase activity, hydrolyzing O-glycosyl compounds
MF	GO:0016798	0.001	hydrolase activity, acting on glycosyl bonds
MF	GO:0015926	0.003	glucosidase activity
MF	GO:0016787	0.030	hydrolase activity
BP	GO:0009816	0.004	defense response to bacterium, incompatible interaction
BP	GO:0009817	0.005	defense response to fungus, incompatible interaction
BP	GO:0042742	0.019	defense response to bacterium
BP	GO:0050832	0.021	defense response to fungus
BP	GO:0009617	0.021	response to bacterium
BP	GO:0005975	0.023	carbohydrate metabolic process
BP	GO:0009814	0.028	defense response, incompatible interaction
BP	GO:0009620	0.029	response to fungus
BP	GO:0045087	0.041	innate immune response
BP	GO:0006955	0.043	immune response
BP	GO:0002376	0.046	immune system process
CC	GO:0005576	0.002	extracellular region

eQTLs for Darkgrey gene modules overlaps with the major Ug99 QTL Rpg-TTKSK locus at 5H. The Darkgrey gene module is enriched with defense related oxidoreductase activity. The Darkgreen module is the only gene module which contains disease resistance genes in adult plants. The major genetic marker for the Darkgreen gene module locates at 7H@83.5cm, which is the major hotspot of differentially expressed genes. The Darkgreen module is enriched with transporter activity, water channel activity, and oxygen binding.

## Discussion

Our prior knowledge guided eQTL mapping method is different from other eQTL mapping methods in three aspects: 1) using data-driven prior knowledge, 2) selecting gene modules for eQTL mapping, 3) identifying major markers for the selected gene modules.

We use the data-driven prior knowledge to identify candidate genes, which is applicable to a wide range of species. Knowledge driven methods, such as Lirnet, are heavily dependent on rich biological knowledge in model organisms. In our method, modules are generated in WGCNA using hierarchical clustering, which is an unsupervised method. Also, QTL and eQTL mapping are performed purely on genotypic, transcript profiling and phenotypic data. Any biological knowledge of these genetic markers and genes are not used in the mapping process. A few studies used biological knowledge in eQTL mapping [5, 8, 9]. Their methods are applicable for resource rich organisms such as yeast, human, mouse and Arabidopsis. For other species, gene functional annotations are very limited.

In our method, selection of candidate genes takes evidence in trait-gene associations and gene-SNP associations. For example, some gene expression values do not have strong associations with traits, such as AF509747.1_at, but have strong associations with SNP markers. Our method is able to find the robust gene modules significantly associated with traits and SNP markers simultaneously.

One of main features of our method is to identify major markers for the selected gene modules. In simulation study, we observe that LassoM and LassoMP are sparse models comparing withe other six models. They used much less number of predictors in the regression models. The major difference between LassoMP and LassoM is LassoMP uses the prior knowledge. The influence of prior knowledge on the performance of eight models in the simulation study is presented in Additional file 3. We proposed to use LassoMP further reduce the number of predictors. LassoMPs is a sparse solution for LassoMP, and the comparison between LassoMPs and LassoMP is available in Additional file 4. From the perspective of model selection, adding prior knowledge reduces the mean-squared error and keep the same proportion of phenotype variance. Moreover, these major regulators are shown to be functionally relevant to rust infection.

## Conclusions

We proposed a new prior knowledge guided eQTL mapping methods for identifying candidate genes. Our method includes three steps: 1) identifying QTLs from QTL mapping; 2) generating and selecting gene modules; 3) prior knowledge guided eQTL mapping. In simulation study, we compared the prior knowledge guided methods LassoMP with other seven multi-task algorithms. The prior knowledge guided eQTL mapping methods outperformed those without prior knowledge. Using LassoMP, in the first barley case study, we identified three gene modules and a few genes as candidate genes for resistance to stem rust, and one of them is confirmed as stem rust resistance gene [10]. In the second case study for stem rust Ug99 resistance in QSM population, we identified four gene modules significantly associated with infection in either seedling or adult plants. One of the gene modules is co-located with the major QTL Rpg-TTKSK for stem rust infection. Another gene module contains four probes mapped to the same disease resistance gene.

The proposed prior knowledge guided eQTL mapping method is applicable for different experimental design and a variety of species. The first case study used all genes and the second case study used a subset of differentially expressed genes on pairwise samples. The identified modules and candidate genes are functionally relevant to rust resistance.

## Additional files

Additional file 1 Enriched GO terms of matched ensemble genes from probe sets in Skyblue module. (PDF 34.2 kb)

Additional file 2 Enriched GO terms of matched ensemble genes from probe sets in Darkgrey and Darkgreen modules. (PDF 35.2 kb)

Additional file 3 Parameter analysis in the simulation study. (PDF 163 kb)

Additional file 4 The comparison between LassoMP and LassoMPs. (PDF 47.9 kb)

## Abbreviations

DEG: Differentially expressed genes
eQTL: Expression quantitative trait loci
IF: Infection frequency
GO: Gene ontology
GWAS: Genome wide association mapping
Lasso: Least absolute shrinkage and selection operator
LES: Lesion size
LOD: Logarithm (base 10) of odds
PC: Principal component
QTL: Quantitative trait loci
SEV: Severity
SNP: Single nucleotide polymorphisms
WGCNA: Weighted gene co-expression network analysis
