# Supplementary material for: Prior knowledge guided eQTL mapping for identifying candidate genes
Source: BMC Bioinformatics. 2016 Dec 13;17:531. doi: 10.1186/s12859-016-1387-9 (PMC5155383; doi:10.1186/s12859-016-1387-9)
Supplement: Additional file 1 — Enriched GO terms of matched ensemble genes from probe sets in Skyblue module. (PDF 34.2 kb) [file 12859_2016_1387_MOESM1_ESM.pdf]

Table 1: Enriched GO terms of matched ensemble genes from probe sets in Skyblue module

GO	GOID	Pvalue	Term
MF	GO:0019144	0.001	ADP-sugar diphosphatase activity
MF	GO:0080041	0.001	ADP-ribose pyrophosphohydrolase activity
MF	GO:0080042	0.001	ADP-glucose pyrophosphohydrolase activity
MF	GO:0004616	0.005	phosphogluconate dehydrogenase (decarboxylating) activity
MF	GO:0004650	0.014	polygalacturonase activity
MF	GO:0048037	0.024	cofactor binding
MF	GO:0003755	0.027	peptidyl-prolyl cis-trans isomerase activity
MF	GO:0016859	0.029	cis-trans isomerase activity
MF	GO:0050661	0.032	NADP binding
BP	GO:0032412	0.001	regulation of ion transmembrane transporter activity
BP	GO:0032409	0.001	regulation of transporter activity
BP	GO:1903959	0.001	regulation of anion transmembrane transport
BP	GO:0034762	0.001	regulation of transmembrane transport
BP	GO:0034765	0.001	regulation of ion transmembrane transport
BP	GO:0022898	0.001	regulation of transmembrane transporter activity
BP	GO:0010359	0.001	regulation of anion channel activity
BP	GO:0043090	0.004	amino acid import
BP	GO:0044070	0.005	regulation of anion transport
BP	GO:0009749	0.009	response to glucose
BP	GO:0098656	0.024	anion transmembrane transport
BP	GO:0009750	0.025	response to fructose
BP	GO:0034284	0.027	response to monosaccharide
BP	GO:0009746	0.027	response to hexose
BP	GO:0000413	0.030	protein peptidyl-prolyl isomerization
BP	GO:0018208	0.030	peptidyl-proline modification
BP	GO:0006865	0.031	amino acid transport
BP	GO:0043269	0.032	regulation of ion transport
BP	GO:0006888	0.034	ER to Golgi vesicle-mediated transport
BP	GO:0051049	0.037	regulation of transport
BP	GO:0006305	0.037	DNA alkylation
BP	GO:0006306	0.037	DNA methylation
BP	GO:0044728	0.038	DNA methylation or demethylation
BP	GO:0006304	0.038	DNA modification
BP	GO:0034285	0.039	response to disaccharide
BP	GO:0009744	0.039	response to sucrose
BP	GO:0055085	0.045	transmembrane transport
BP	GO:0032879	0.045	regulation of localization
BP	GO:0015849	0.048	organic acid transport
BP	GO:0046942	0.048	carboxylic acid transport
CC	GO:0009570	0.031	chloroplast stroma
CC	GO:0009532	0.033	plastid stroma
