# Supplementary material for: Prior knowledge guided eQTL mapping for identifying candidate genes
Source: BMC Bioinformatics. 2016 Dec 13;17:531. doi: 10.1186/s12859-016-1387-9 (PMC5155383; doi:10.1186/s12859-016-1387-9)
Supplement: Additional file 2 — Enriched GO terms of matched ensemble genes from probe sets in Darkgrey and Darkgreen modules. (PDF 35.2 kb) [file 12859_2016_1387_MOESM2_ESM.pdf]

Table 1: Enriched GO terms of matched ensemble genes from probe sets in Darkgrey module

GO	GOID	Pvalue	Term
MF	GO:0004351	0.002	glutamate decarboxylase activity
MF	GO:0030145	0.012	manganese ion binding
MF	GO:0045735	0.015	nutrient reservoir activity
MF	GO:0016831	0.018	carboxy-lyase activity
MF	GO:0004252	0.029	serine-type endopeptidase activity
MF	GO:0016830	0.029	carbon-carbon lyase activity
MF	GO:0042802	0.030	identical protein binding
MF	GO:0030170	0.032	pyridoxal phosphate binding
MF	GO:0004601	0.039	peroxidase activity
MF	GO:0016684	0.039	oxidoreductase activity, acting on peroxide as acceptor
MF	GO:0017171	0.046	serine hydrolase activity
MF	GO:0016209	0.046	antioxidant activity
MF	GO:0008236	0.046	serine-type peptidase activity
BP	GO:0006536	0.008	glutamate metabolic process
BP	GO:0043648	0.023	dicarboxylic acid metabolic process
BP	GO:0009064	0.024	glutamine family amino acid metabolic process
BP	GO:0043086	0.034	negative regulation of catalytic activity
BP	GO:0044092	0.035	negative regulation of molecular function

Table 2: Enriched GO terms of matched ensemble genes from probe sets in Darkgreen module

GO	GOID	Pvalue	Term
MF	GO:0015200	0.002	methylammonium transmembrane transporter activity
MF	GO:0005275	0.003	amine transmembrane transporter activity
MF	GO:0008519	0.003	ammonium transmembrane transporter activity
MF	GO:0005215	0.004	transporter activity
MF	GO:0008509	0.004	anion transmembrane transporter activity
MF	GO:0015101	0.005	organic cation transmembrane transporter activity
MF	GO:0015250	0.006	water channel activity
MF	GO:0005372	0.006	water transmembrane transporter activity
MF	GO:0016843	0.008	amine-lyase activity
MF	GO:0016844	0.008	strictosidine synthase activity
MF	GO:0019825	0.016	oxygen binding
MF	GO:0015171	0.019	amino acid transmembrane transporter activity
MF	GO:0004650	0.024	polygalacturonase activity
MF	GO:0016840	0.024	carbon-nitrogen lyase activity
MF	GO:0051537	0.028	2 iron, 2 sulfur cluster binding
MF	GO:0009055	0.032	electron carrier activity
MF	GO:0015075	0.034	ion transmembrane transporter activity
MF	GO:0015103	0.036	inorganic anion transmembrane transporter activity
MF	GO:0046943	0.038	carboxylic acid transmembrane transporter activity
MF	GO:0005342	0.038	organic acid transmembrane transporter activity
MF	GO:0022838	0.048	substrate-specific channel activity
MF	GO:0015267	0.050	channel activity
MF	GO:0022803	0.050	passive transmembrane transporter activity
MF	GO:0008514	0.050	organic anion transmembrane transporter activity
BP	GO:0098656	0.001	anion transmembrane transport
BP	GO:0071705	0.001	nitrogen compound transport
BP	GO:0033331	0.002	ent-kaurene metabolic process
BP	GO:0015843	0.002	methylammonium transport
BP	GO:0072488	0.002	ammonium transmembrane transport
BP	GO:0072489	0.002	methylammonium transmembrane transport
BP	GO:0015696	0.002	ammonium transport
BP	GO:0010241	0.002	ent-kaurene oxidation to kaurenoic acid
BP	GO:0015837	0.004	amine transport
BP	GO:0015695	0.004	organic cation transport
BP	GO:0016101	0.005	diterpenoid metabolic process
BP	GO:0016102	0.005	diterpenoid biosynthetic process
BP	GO:0009685	0.005	gibberellin metabolic process
BP	GO:0009686	0.005	gibberellin biosynthetic process
BP	GO:0019755	0.005	one-carbon compound transport
BP	GO:0098661	0.007	inorganic anion transmembrane transport
BP	GO:0071370	0.009	cellular response to gibberellin stimulus
BP	GO:0009740	0.009	gibberellic acid mediated signaling pathway
BP	GO:0010476	0.009	gibberellin mediated signaling pathway
BP	GO:0034220	0.011	ion transmembrane transport
BP	GO:0042214	0.011	terpene metabolic process
BP	GO:0009739	0.011	response to gibberellin
BP	GO:0006820	0.014	anion transport
BP	GO:0003333	0.021	amino acid transmembrane transport
BP	GO:1903825	0.028	organic acid transmembrane transport
BP	GO:0015833	0.030	peptide transport
BP	GO:0006857	0.030	oligopeptide transport
BP	GO:0042886	0.034	amide transport
BP	GO:0042044	0.042	fluid transport
BP	GO:0006833	0.042	water transport
CC	GO:0042807	0.002	central vacuole
CC	GO:0000322	0.002	storage vacuole
CC	GO:0000326	0.002	protein storage vacuole
CC	GO:0009707	0.010	chloroplast outer membrane
CC	GO:0009705	0.012	plant-type vacuole membrane
CC	GO:0009527	0.015	plastid outer membrane
CC	GO:0005732	0.017	small nucleolar ribonucleoprotein complex
CC	GO:0000325	0.019	plant-type vacuole
CC	GO:0031968	0.043	organelle outer membrane
CC	GO:0009505	0.045	plant-type cell wall
