# Supplementary material for: Prior knowledge guided eQTL mapping for identifying candidate genes
Source: BMC Bioinformatics. 2016 Dec 13;17:531. doi: 10.1186/s12859-016-1387-9 (PMC5155383; doi:10.1186/s12859-016-1387-9)
Supplement: Additional file 3 — Parameter analysis in the simulation study. (PDF 163 kb) [file 12859_2016_1387_MOESM3_ESM.pdf]

The Influence of prior knowledge on the performance of LassoMP, RidgeMP and elasticMP in four setups are presented in Figure 1. The prior knowledge is represented as the penalty factor $p_j \in [0, 1]$ for SNP, 0 means no penalty on the predictor, and 1 means a full penalty on the predictor. No penalty on LassoMP, RidgeMP and elasticMP reaches the lowest RMSE. It means prior knowledge is useful to reduce the errors in these three models. The best AUC is reached in weaker or full penalty in RidgeMp and elasticMP. It means that the influence of the penalty factor on the RMSE and AUC is opposite. In terms of DF, the penalty factor almost has no impact on RidgeMP and elasticMP, and small influence on LassoMP.

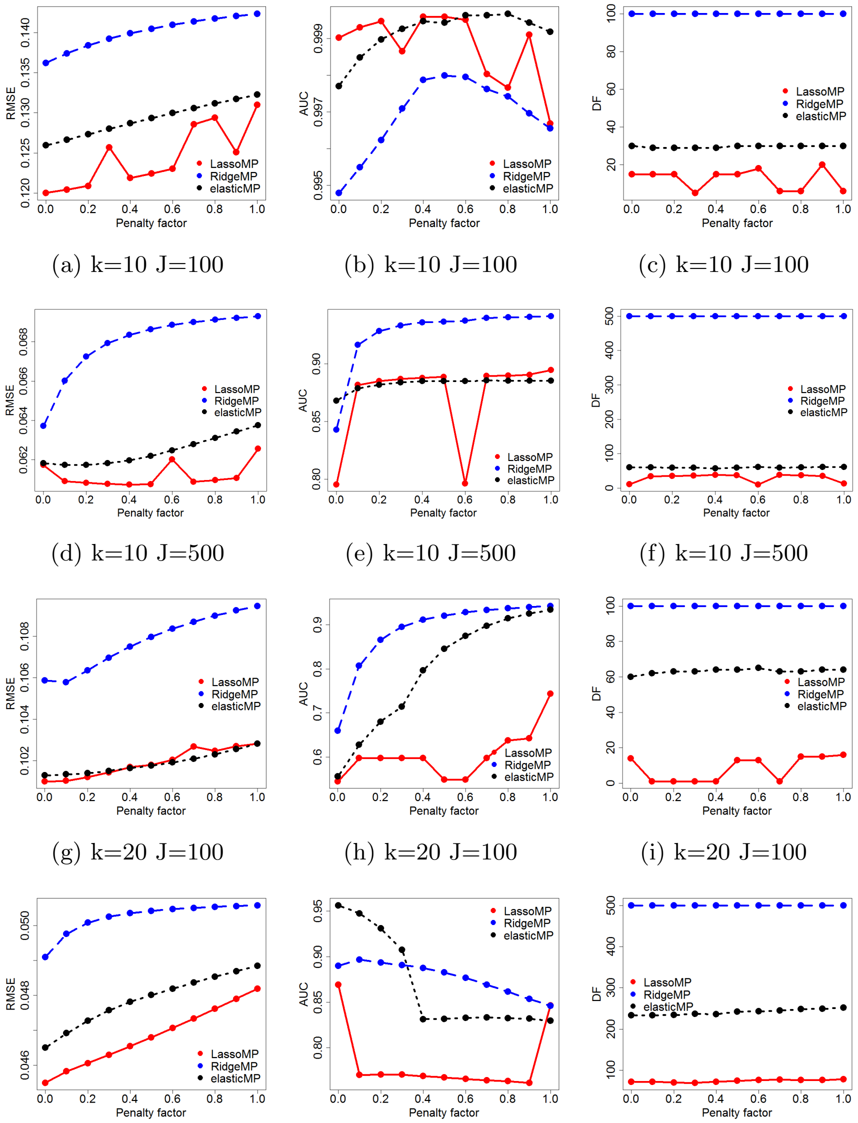


(a) k=10 J=100 (b) k=10 J=100 (c) k=10 J=100

(d) k=10 J=500 (e) k=10 J=500 (f) k=10 J=500

(g) k=20 J=100 (h) k=20 J=100 (i) k=20 J=100

(j) k=20 J=500 (k) k=20 J=500 (l) k=20 J=500

Figure 1: The influence of penalty factor on performance measures in simulation study
