# Supplementary material for: Prior knowledge guided eQTL mapping for identifying candidate genes
Source: BMC Bioinformatics. 2016 Dec 13;17:531. doi: 10.1186/s12859-016-1387-9 (PMC5155383; doi:10.1186/s12859-016-1387-9)
Supplement: Additional file 4 — The comparison between LassoMP and LassoMPs. (PDF 47.9 kb) [file 12859_2016_1387_MOESM4_ESM.pdf]

LassoMs is a sparse solution based on the same regression model as LassoM. We use letter "s" to represent the corresponding sparse solution. In the same manner, we can derive LassoMPs from LassoMP. We compared LassoM, LassoMs, LassoMP and LassoMPs in the first barley case study.

We compared LassoMs with LassoM, and LassoMPs with LassoMP with measures in model selection and the biological meaning (Table 4). LassoMs and LassoMPs use the same objective function as LassoM and LassoMP, but they are the most regularized models within one standard error of minimum Lasso and LassoMP respectively. In glmnet, multi-response Gaussian family with $\lambda = lambda.min$ is used for LassoM, and multi-response Gaussian family with $\lambda = lambda.1se$ is used for LassoMs. In the similar way, LassoMP and LassoMPs can be obtained by setting the parameter penalty.factor on LassoM and LassoMs. LassoMs and LassoMPs include a much lower number of predictors, slightly higher MSE, and lower proportion of variance explained compared to LassoM and LassoMP. LassoMPs only includes at most three predictors in these three gene modules. LassoMPs has the highest proportion of cis eQTLs among these four methods.

LassoMP is the best model in terms of model selection, and LassoMPs is the most sparse model with high proportion of cis eQTLs. In real applications, LassoMPs is a good choice for a practical solution in eQTL mapping since LassoMPs keep the most significant genetic markers in the regression model.

Table 1: The comparison of four methods on three gene modules

Gene modules	Method	Mean Squared Error	# of predictors	Proportion of variance explained(%)	# of eQTLs	# of eQTLs with known gene locations	Proportion of cis eQTLs (%)
Plum1	LassoM	1750411	23	56.01	621	69	17.39
	LassoMP	1639614	9	52.13	243	27	29.63
	LassoMs	1903784	6	48.06	162	18	77.78
	LassoMPs	1735975	1	45.26	27	3	100.00
Skyblue	LassoM	2918151	47	52.65	1269	47	9.57
	LassoMP	2801753	2	36.68	54	4	50.00
	LassoMs	3161290	5	36.36	135	10	90.00
	LassoMPs	2802788	1	36.43	27	2	100.00
Saddlebrown	LassoM	2919870	14	46.73	378	28	21.43
	LassoMP	2675651	3	47.23	81	21	100.00
	LassoMs	3187274	3	40.84	81	21	100.00
	LassoMPs	2675651	3	47.23	81	21	100.00
